# Improving mental health outcomes: achieving equity through quality improvement

**DOI:** 10.1093/intqhc/mzu005

**Published:** 2014-02-11

**Authors:** Alan J. Poots, Stuart A. Green, Emmi Honeybourne, John Green, Thomas Woodcock, Ruth Barnes, Derek Bell

**Affiliations:** 1 NIHR CLAHRC for Northwest London, Imperial College London, Chelsea and Westminster Hospital, 369 Fulham Road, London SW10 9NH, UK; 2 Westminster IAPT Primary Care Psychology Service, 192–198 Vauxhall Bridge Road, London SW1V 1DX, UK; 3 Department of Clinical Health Psychology, St Mary's Hospital, Praed Street, London W2 1NY, UK; 4 NHS Northwest London, Marylebone Road, London, UK

**Keywords:** quality improvement, mental health, public health, inequalities, outcome assessment (health care)

## Abstract

**Objective:**

To investigate equity of patient outcomes in a psychological therapy service, following increased access achieved by a quality improvement (QI) initiative.

**Design:**

Retrospective service evaluation of health outcomes; data analysed by ANOVA, chi-squared and Statistical Process Control.

**Setting:**

A psychological therapy service in Westminster, London, UK.

**Participants:**

People living in the Borough of Westminster, London, attending the service (from either healthcare professional or self-referral) between February 2009 and May 2012.

**Intervention(s):**

Social marketing interventions were used to increase referrals, including the promotion of the service through local media and through existing social networks.

**Main Outcome Measure(s):**

(i) Severity of depression on entry using Patient Health Questionnaire-9 (PHQ9). (ii) Changes to severity of depression following treatment (ΔPHQ9). (iii) Changes in attainment of a meaningful improvement in condition assessed by a key performance indicator.

**Results:**

Patients from areas of high deprivation entered the service with more severe depression (*M* = 15.47, SD = 6.75), compared with patients from areas of low (*M* = 13.20, SD = 6.75) and medium (*M* = 14.44, SD = 6.64) deprivation. Patients in low, medium and high deprivation areas attained similar changes in depression score (ΔPHQ9: *M* = −6.60, SD = 6.41). Similar proportions of patients achieved the key performance indicator across initiative phase and deprivation categories.

**Conclusions:**

QI methods improved access to mental health services; this paper finds no evidence for differences in clinical outcomes in patients, regardless of level of deprivation, interpreted as no evidence of inequity in the service with respect to this outcome.

## Introduction

In the UK, the Department of Health explicitly includes policy to reduce health inequalities within the National Health Service (NHS). Over the last 30 years much of the policy focus has been on the wider determinants of health but more recent efforts have included improving access to services [1]. National policy recognizes inequities in access lead to poorer clinical outcomes and exacerbate health inequalities; thus improving equal access on the basis of need should be a priority [2]. Improving access to mental health services is an example where national policy has been developed to address inequities. The Improving Access to Psychological Therapies (IAPT) programme is based on evidence-based recommendations from the National Institute of Health and Clinical Excellence, proposing cognitive behavioural therapy (and additional specific therapies) should be available for a range of common mental disorders (CMDs), including anxiety and depression [3–7]. The IAPT programme aims to improve access to treatment by providing an accessible community-based service that improves clinical outcomes for patients, delivering a more equitable service [8].

Specific barriers to improving access for ‘seldom-heard groups’ have been identified, recognizing that certain groups are less likely to access appropriate services, e.g. those with medically unexplained symptoms and people from black and minority ethnic groups [9, 10]. Improving access to mental health services cannot be achieved by ‘one-size-fits-all’ approaches; the IAPT programme allows providers to develop locally relevant strategies aligned to the needs of local populations.

Like many long-term conditions, CMDs are associated with deprivation [11], measured in the UK by the Index of Multiple Deprivation (IMD). Improving access to services is an important step in delivering equitable services, and should be accompanied by evidence of improvements in clinical outcomes.

The Westminster IAPT Primary Care Psychology Service was introduced in February 2009, and worked in partnership with the National Institute for Health Research (NIHR) Collaboration for Leadership in Applied Health Research and Care (CLAHRC) Northwest London, a quality improvement (QI) support programme, to increase referrals to the service between April 2010 and September 2011.

In the Westminster IAPT initiative, the QI approach facilitated the distribution of social marketing materials to increase awareness of the service, accompanied by a staged introduction of a self-referral route. The initiative aimed to increase referrals to the service, especially from people in deprived areas. A geospatial evaluation demonstrated that the strategy improved access to the service for patients from more deprived areas [12]. This paper builds on that analysis, assessing outcomes over time and across different categories of deprivation, as a measure of equity.

## Methods

### Quality improvement

NIHR CLAHRC Northwest London was established in 2008 as one of nine CLAHRCs in England, created to address the slow rate of implementation and spread of research findings in the NHS: the ‘second translational gap’ [13]. NIHR CLAHRC Northwest London assists clinical teams in the application of a comprehensive package of QI methods to support the sustainable implementation of evidence-based interventions. A central method is the ‘Model for Improvement’ [14], providing a framework to delivering an intervention and monitoring its successful uptake.

### Social marketing and self-referral interventions

The Westminster IAPT worked to increase referrals to their service using QI methods, including weekly collection of referral data and small-scale tests of change (Plan-Do-Study-Act cycles) to assess the phased delivery of social marketing interventions including:

- Distribution of leaflets and posters across public and community venues throughout the borough.
- Multi-media promotion of the service, including local radio, local newspapers and online.
- Accessing social networks (e.g. community groups, churches, societies and public and voluntary organizations) to encourage referral.

### Data sources

Routine demographic and clinical data from all patients referred to the service were collected as per the minimum data set schedule using the IAPTus (Mayden, Wiltshire, UK) clinical data system [15]. Anonymized geocoded data were extracted for residents of the London borough of Westminster referred to the service during the period of analysis, February 2009 and May 2012, in line with a previous analysis [12]. The following data were extracted for each patient; square parentheses show correspondence to IAPT minimum data standard:

- Lower Super Output Area (LSOA) [derived from person item 7].
- Date referral received [referral item 16].
- Date of initial assessment [derived from appointment item 25].
- Date of first therapeutic session [derived from appointment item 25].
- Number of attended sessions [count of appointments].
- Reason for end of IAPT care pathway [referral item 23].
- Patient Health Questionnaire-9 (PHQ9) at first session [derived from appointment item 37].
- PHQ9 at last session [derived from appointment item 37].

Inclusion criteria:

- Patient referred to IAPT Westminster service between 28 February 2009 and 21 May 2012.
- LSOA of patients' residence falls within London Borough of Westminster boundary.

### Data processing

Removal of duplicates from the extracted data based on postcode, date of initial assessment and reference number was performed. Data were aggregated into ‘baseline’, ‘implementation’ or ‘sustainability’ phases relating to the existence of the QI collaborative tasked with improvement:

- Baseline: service running prior to the establishment of the QI collaborative. Week 1 (February 2009) through to Week 58 (April 2010; 58 weeks).
- Implementation: service running post-establishment of the QI collaborative. Week 59 (April 2010) to Week 135 (September 2011, 77 weeks).
- Sustainability: service running post-dis-establishment of the QI collaborative. Week 136 (September 2011) to Week 170 (May 2012, 35 weeks).

To maintain confidentiality, the postcodes for each patient were assigned to corresponding LSOA [16] by the clinical team, using an online geospatial tool GeoConvert (http://geoconvert.mimas.ac.uk). These governmental geographical regions are fine resolution and many UK statistics are produced at this level, including IMD, enabling attribution of an estimate of socio-economic deprivation to each record; these were categorized by quintiles: quintile 1, low deprivation; quintiles 2–4, medium deprivation; quintile 5, high deprivation.

### Outcome measures

The patient outcomes from the Westminster IAPT initiative at a population level are evaluated, using a measure of severity of depression (PHQ9), recorded as standard in the IAPT data set. A modified key performance indicator (KPI) [15, 17]—Move To Recovery (MTR)—based on PHQ9 scores is designed to evaluate attainment of a meaningful improvement in severity of depression.

The outcome measures considered in this paper are designed to assess

1. the severity of depression on entry, those records with a recorded PHQ9;
2. changes to the severity of depression using ΔPHQ9 (the change in score between entry and exit to the service for those patients with a planned exit—agreement between patient and clinician);
3. changes in attainment of a meaningful improvement in condition, as measured by MTR. Here this is restricted to a measure solely concerned with the depression scores on entry and exit (MTRDEP). To achieve MTRDEP, PHQ9 on entry to the IAPT service must be equal to or higher than 10 and less than 10 on a planned exit from the service, with 10 indicative of ‘caseness’—the patient is considered to have at least mild severity depression.

## Analysis

### Analysis of severity of depression on entry

Two-way ANOVA compares the mean PHQ9 on entry for patients from low, medium and high levels of deprivation during the baseline, implementation and sustainability phases of the QI initiative. The normality and homoscedasticity assumptions of ANOVA were tested.

### Analysis of change in depression score between entry and exit

Two-way ANOVA compares the mean ΔPHQ9 for patients from low, medium and high levels of deprivation during the baseline, implementation and sustainability phases of the QI initiative. The normality and homoscedasticity assumptions of ANOVA were tested.

### Analysis of effectiveness of a KPI to demonstrate clinical outcome

Statistical process control (SPC, see Box 1) was used to assess whether there were changes in the proportion of patients attaining MTRDEP. In this case, a p-chart is used, since the measure is a proportion of patients satisfying certain criteria and the denominator is not constant from month to month [18].

Box 1 **Key notes for SPC**

∙ SPC was developed by Shewhart in the 1920s [18] as a statistical methodology for detecting changes in time-series data through quantification of the natural variation exhibited in any system, without explicit knowledge of the underlying distribution [23, 24]. Its use in healthcare has increased over the last 20 years and can be useful in managing change [25].

∙ SPC is an appropriate and flexible analysis of time-series data, assessing for changes in process.

∙ SPC uses a variety of control charts depending on the situation, all of which plot the data, a measure of central tendency (often the mean) and two 3-sigma control limits demarking the expected variation of the system. These are typically displayed as four lines—one showing the data, one a solid line of central tendency and two dashed control limits.

∙ These features are used to diagnose properties of the system, using well-established rules to determine whether the system is stable including [23]

∘ a point lying outside either of the control limits;

∘ a run of seven consecutive points all above or all below the central line;

∘ a run of seven consecutive points all increase or all decrease.

∙ If a ‘rule break’ has occurred, the system is not stable and is changing—it is ‘out of control’.

∙ This ‘out-of-control’ state is desired, at least in the short term, if one is trying to alter the system into a new mode of operation, i.e. improve from a stable underperforming system to a better performing one.

Three chi-squared tests, one for each initiative phase, were used to examine for effect of IMD category on the numbers attaining MTRDEP. In each case, the null hypothesis states that the proportion of patients achieving MTR does not differ across categories of deprivation.

## Results

During the period of analysis, February 2009 to May 2012, 6062 patients were referred to the IAPT service. During the baseline phase, Weeks 1–58 (February 2009 to March 2010), before the start of the QI initiative, 992 referrals were made with a mean referral of 17.02 (SD = 14.44) patients per week; the implementation phase, Weeks 59–135 (April 2010 to September 2011), saw 3295 patients referred with an mean referral of 42.69 (SD = 12.46) patients per week; finally the sustainability phase, Weeks 136–170 (October 2011 to May 2012) saw a total of 1791 patients referred with a mean weekly referral of 51.09 (SD = 13.13) patients per week. A one-way ANOVA determines a significant difference in the means of referrals between the periods (*F*(2167) = 91.254, *P* < 0.001). A Tukey *post hoc* test revealed that mean referrals in the baseline (*M* = 17.02, SD = 14.44) were significantly lower than for implementation (*M* = 42.69, SD = 12.46, *P* < 0.001) and sustainability (*M* = 51.09, SD = 13.13, *P* < 0.001) phases, which themselves were statistically different (*P* = 0.006).

Patients included were referred from 120 unique LSOAs. Of the 6062, 3864 (64%) patients had a PHQ9 recorded for first assessment; 1599/6062 (26%) had a planned exit from the service (agreement between patient and clinician).

Both first and last PHQ9 scores were recorded for 1426/6062 (24%), enabling the calculation of ΔPHQ9. First PHQ9 scores of >10—‘caseness’—were found for 1003/1426 (70%), of whom 662/1003 (66%) achieved MTRDEP. The percentages of patients not informed of other services or had an unplanned or exit (i.e. ‘dropping-out’) were 450/987 (46%) in the baseline phase, 1552/3287 (47%) in the implementation phase and 1206/1788 (67%) in the sustainability phase. A higher rate of dropout was observed in the high deprivation areas (841/1428, 59%) compared with the low deprivation (459/955, 48%) and medium deprivation (1908/3679, 52%) areas. Chi-squared tests of these proportions find statistically significant differences in drop-out rates with regard to period [*χ*^2^ (2, *N* = 6062) = 215.69, *P* < 0.001] and IMD category [*χ*^2^ (2, *N* = 6062) = 31.14, *P* < 0.001].

### Analysis of severity of depression on entry

**Figure 1:**
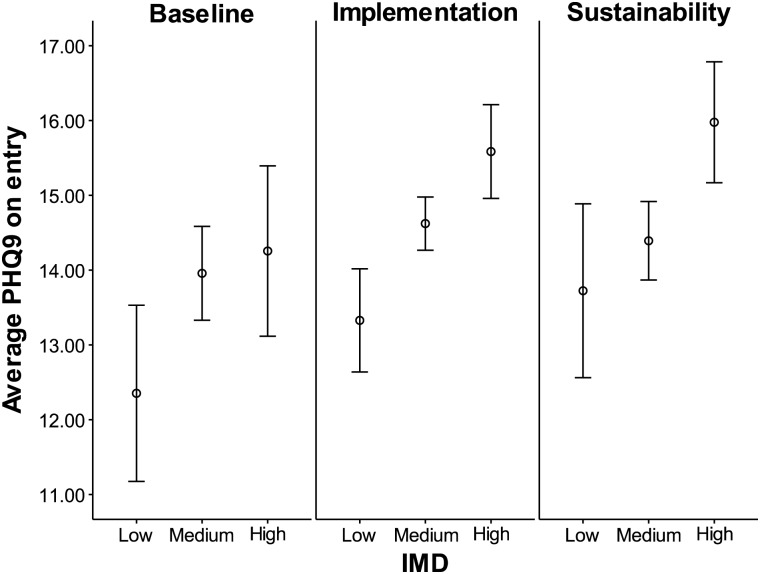
The average PHQ9 on entry for patients. A higher value represents a greater severity of depression. This figure shows that both IMD and initiative phase impact the average PHQ9 score at entry. 95% confidence intervals are plotted.

Two-way ANOVA finds deprivation, as measured by IMD category (*F*(2,3855) = 15.06, *P* = 0.004), and initiative phase (*F*(2,3855) = 5.58, *P* < 0.001) have a statistically significant impact on the PHQ9 on entry to the service, but the interaction between these variables is not significant (*F*(4,3855) = 0.77, *P* = 0.54). The average values are plotted in Fig. 1: patients from high IMD areas (i.e. high deprivation areas) attend the service with a higher PHQ9 score (*M* = 15.47, SD = 6.75) indicative of a more severe level of depression, compared with low (*M* = 13.20, SD = 6.75) and medium (*M* = 14.44, SD = 6.64) IMD areas. Tukey *post hoc* tests reveal significant differences between low and medium (*P* < 0.001); low and high (*P* < 0.001) and medium and high (*P* < 0.001) pairings. The mean PHQ9 on entry in both the implementation phase (*M* = 14.61, SD = 6.71) and the sustainability phase (*M* = 14.71, SD = 6.63) is higher than that in the baseline phase (*M* = 13.72, SD = 6.83). Tukey *post hoc* tests reveal significant differences between baseline and implementation (*P* = 0.005) and baseline and sustainability (*P* = 0.007) pairings, but no difference for implementation and sustainability (*P* = 0.921).

### Analysis of change in depression score between entry and exit

**Figure 2:**
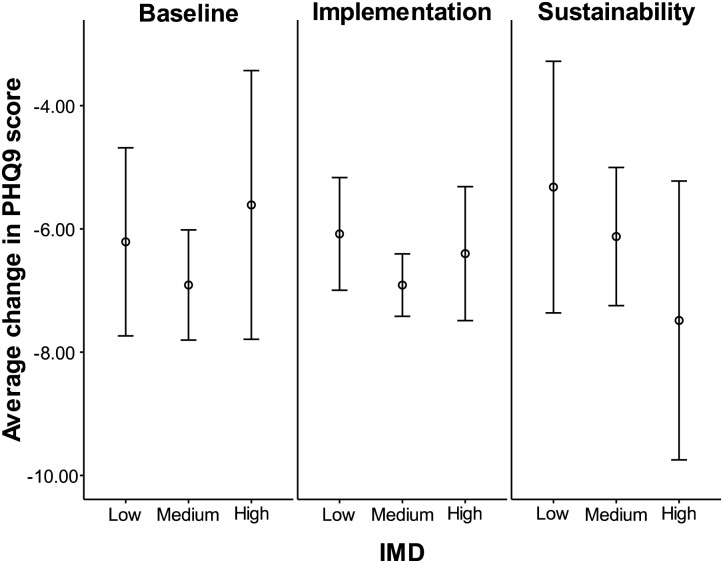
The average ΔPHQ9 for patients with planned exit. A negative value represents an improvement. This figure shows that both IMD and initiative phase do not affect the average ΔPHQ9 score. 95% confidence intervals are plotted.

Two-way ANOVA finds no significant effect of IMD category (*F*(2,1417) = 0.90, *P* = 0.406), initiative phase (*F*(2,1417) = 0.11, *P* = 0.894) or the interaction term (*F*(4,1417) = 0.77, *P* = 0.543) on the average ΔPHQ9. The average values are plotted in Fig. 2. The null hypothesis of no difference between the average values as classified by the initiative phase and IMD cannot be rejected.

This result finds no evidence that the initiative phase (and thus intensity of referrals, as more referrals occurred in the implementation and sustainability phases) and deprivation affect the benefit that patients received from the service (ΔPHQ9: *M* = −6.60, SD = 6.41). However, Fig. 2 shows some differences in patterns comparing the sustainability phase with the other phases.

### Analysis of effectiveness of a KPI to demonstrate clinical outcome

**Figure 3:**
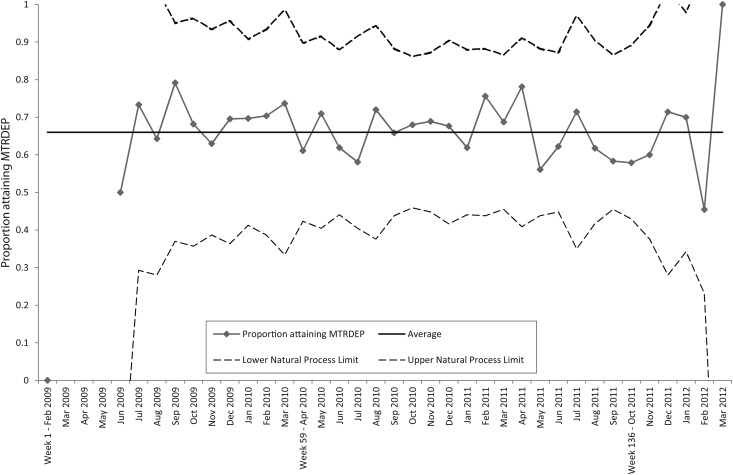
A p-chart showing the proportion attaining MTRDEP, at monthly frequency and with variable width limits of expected variation, accounting for differing denominators. No rule breaks were observed. The process is considered to be in statistical control.

SPC analysis does not detect any ‘rule breaks’ (see Box 1) for changes in process in the proportion of patients attaining MTRDEP (Fig. 3), implying a stable process. Thus, 66% of these exits are expected to achieve MTRDEP on average, implying that regardless of intensity of referral there is no evidence in the data for service inequity by deprivation category.

None of the chi-squared tests of interaction can reject the null hypothesis of no association between project period and the proportions attaining MTRDEP in each IMD category [baseline: *χ*^2^ (2, *N* = 209) = 1.91, *P* = 0.38; implementation: *χ*^2^ (2, *N* = 676) = 1.61, *P* = 0.44; sustainability: *χ*^2^ (2, *N* = 118) = 3.74, *P* = 0.15)]. Thus, during each initiative phase there is no evidence for inequity of outcome in this measure.

## Discussion

During the implementation phase, the average weekly referral rate increased compared with the baseline phase; which was subsequently sustained. It is difficult to associate any specific component of the intervention with increases in referrals due to the concurrent delivery of several components; yet taken as a whole, the combined intervention demonstrated an increase in referral.

A previous geospatial evaluation demonstrated that a strategy to improve access to the Westminster IAPT service increased access for all patients, especially from more deprived areas with associated higher healthcare needs. [9] This analysis of the same cohort of patients finds no evidence for non-equivalence of clinical outcomes between areas of differing levels of deprivation.

Patients entering the service from areas of higher deprivation have a higher average PHQ9 score; this gives credence to an underlying assumption that the severity of CMDs, specifically depression, is positively associated with IMD score, which acts as a proxy for need. In addition, the initiative phase seems to be important, with the people accessing the service having higher PHQ9 scores on average during the implementation and sustainability phases than in the baseline phase, and there was no overall tendency for the service to be accepting referrals from the ‘worried well’. Whilst differences in PHQ9 scores exist for entry, the clinical meaningfulness of this is doubtful.

No evidence for inequity of outcome between areas of differing levels of deprivation is observed in these data between the phases. Therefore, even with increased access to the service because of the QI initiative, there is no evidence that the prescribed course of therapies benefited patients from all deprivation categories differentially.

The analysis of MTRDEP, demonstrating meaningful changes in depression, found no difference between patients living in areas of low, medium and high deprivation or with the phases of the QI initiative. Whilst there are more dropouts in the sustainability phase, the QI initiative aimed only to increase access; this finding highlights a novel issue that those people now encouraged to join might be less likely to be retained. Analysis of the point at which dropout occurs over the prescribed course of treatment could be considered, and used to aid retention.

In Europe, the odds of people from more disadvantaged backgrounds (i.e. low educational attainment, low income and lower occupational status) suffering from CMDs have been estimated to be 1.5–2 times the rest of the population [11]. There can be significant levels of unmet mental health need in communities, especially in inner cities, where demographics and social factors affect consultation and health-seeking behaviour [10], requiring health policies and initiatives need to tackle these inequalities at both local and national levels.

Overall, 66% of patients with planned exits ‘moved to recovery’, across time and categories of deprivation. Whilst some patients did not achieve a ‘meaningful clinical outcome’ (as defined by the KPI), they may still benefit from access to the service, as mental health is often linked to other long-term needs. Physical and mental health has a complex relationship, and so providing care to those with long-term needs can have other benefits not captured by PHQ9 metrics.

This analysis provides knowledge of a healthcare system at a population level and highlights the use of routine data in providing decision support and service evaluation. The importance of using data that are currently available and pertinent to the service should not be overlooked: they provide an opportunity to evaluate with minimal burden to service staff and generate outputs with metrics familiar to providers and commissioners alike [19]. This is particularly beneficial where metrics are available at high temporal and geographical resolutions, allowing processes and outcomes of care to be monitored.

### Limitations of this study

The outcomes for those living within areas of low, medium and high deprivation have no evidence of inequity, but there is heterogeneity in outcome at a patient level: within each group, some patients are responding more to treatment. A patient level model encompassing demographics and finer scale predictors would be required to investigate this heterogeneity. The anxiety component of an MTR metric was not considered: the measure of anxiety (GAD7) has not yet had its responsiveness over time directly evidenced as a primary measure in longitudinal studies [20]. Hence, the depression metric (PHQ9), which has been so evidenced, was used [21]. A single self-report measure (PHQ9) may not capture the full complexity of each case, but as the prescribed therapies have been evidence to affect this measure directly [4, 6, 21] and it is the recorded clinical outcome, this analysis focuses on the depression score.

Whilst a previous geographic analysis showed improved equity of access [12], and here no evidence of differences in outcomes is found (at a population level) for those completing the prescribed course of therapies, there are differences in the proportions dropping out of the service from high, medium and low deprivation areas. Research into factors affecting dropout at a patient level would be useful to examine for any differential exits. Finally, this analysis examines the outcomes of all referral types, as there is a difficulty in separating self-referrals from GP initiated self-referrals, because the IAPT data standard does not hold this information.

## Conclusion

This analysis found no evidence of differing clinical outcomes for patients in a local IAPT service from areas of different levels of deprivation. At a population level, throughout time, no evidence was found for influences of increased input. To explore factors that may influence clinical outcome, a patient level analysis with socio-demographic variables in a predictive model would be helpful to elucidate any heterogeneity masked at a population level.

The UK's Department of Health holds a vision of a patient-led healthcare system that delivers better health outcomes, with local autonomy and accountability. QI provides a methodology to ensure service providers are responsive to the needs of local populations for the delivery of equitable care. Aligning QI to population health can help to demonstrate how service improvements have affected the population as a whole, or to identify priorities for improvement [22].

## Ethical approval

Ethical approval was not required for this work as it is part of a service evaluation and improvement project.

## Funding

This work was supported by the National Institute for Health Research (NIHR) Collaboration for Leadership in Applied Health Research and Care (CLAHRC) for Northwest London and the Central and North West London NHS Foundation Trust. This article presents independent research commissioned by the National Institute for Health Research (NIHR) under the Collaborations for Leadership in Applied Health Research and Care (CLAHRC) programme for North West London. The views expressed in this publication are those of the author(s) and not necessarily those of the NHS, the NIHR or the Department of Health. Funding to pay the Open Access publication charges for this article was provided by Imperial College London.
